# Feasibility of a Mobile App–Based Cognitive-Behavioral Perinatal Skills Program: Protocol for Nonrandomized Pilot Trial

**DOI:** 10.2196/59461

**Published:** 2025-01-28

**Authors:** Andrea B Temkin-Yu, Aliza Ayaz, Ella Blicker, Michael X Liu, Ace Oh, Isabelle E Siegel, Meredith J Seewald, Alison D Hermann, Soudebah Givrad, Lara M Baez, Lauren M Osborne, Cori M Green, Maddy M Schier, Alexandra M Davis, Shasha Zhu, Avital Falk, Shannon M Bennett

**Affiliations:** 1 Psychiatry Department Weill Cornell Medicine New York, NY United States; 2 Preventive Medicine Feinberg School of Medicine Northwestern University Evanston, IL United States; 3 Clinical Psychology at Palo Alto University Psychiatry at Stanford Medicine Palo Alto, CA United States; 4 Psychology Department Florida International University Miami, FL United States

**Keywords:** perinatal mood and anxiety disorders, apps, smartphones, digital intervention, cognitive behavioral therapy, feasibility, pilot trial, mobile phones

## Abstract

**Background:**

Mental illness is one of the top causes of preventable pregnancy-related deaths in the United States. There are many barriers that interfere with the ability of perinatal individuals to access traditional mental health care. Digital health interventions, including app-based programs, have the potential to increase access to useful tools for these individuals. Although numerous mental health apps exist, there is little research on developing programs to address the unique needs of perinatal individuals. In an effort to fill this gap, a multidisciplinary team of experts in psychology, psychiatry, obstetrics, and pediatric primary care collaborated to develop the novel Perinatal Skills Program within Maya, a flexible and customizable cognitive-behavioral skills app. Maya-Perinatal Skills Program (M-PSP) uses evidence-based strategies to help individuals manage their mood and anxiety symptoms during pregnancy and post partum.

**Objective:**

This pilot study aims to assess the feasibility, acceptability, and usability of M-PSP and explore links between program use and symptoms of anxiety and low mood.

**Methods:**

This single-arm trial will recruit 50 pregnant or postpartum individuals with mild-to-moderate anxiety or mood symptoms. Participants will be recruited from a variety of public and private insurance-based psychiatry, obstetrics, and primary care clinics at a large academic medical center located in New York City. Participants will complete all sessions of M-PSP and provide feedback. Outcome measures will include qualitative and quantitative assessments of feasibility, acceptability, and usability, passively collected program usage data, and symptom measures assessing mood, anxiety, and trauma. Planned data analysis includes the use of the grounded theory approach to identify common themes in qualitative feedback, as well as an exploration of possible associations between quantitative data regarding program use and symptoms.

**Results:**

The recruitment began on August 2023. As of October 2024, a total of 32 participants have been enrolled. The recruitment will continue until 50 participants have been enrolled.

**Conclusions:**

Digital health interventions, like M-PSP, have the potential to create new pathways to reach individuals struggling with their mental health. The results of this study will be the groundwork for future iterations of M-PSP in the hopes of providing an accessible and helpful tool for pregnant and postpartum individuals.

**Trial Registration:**

ClinicalTrials.gov NCT05897619; https://classic.clinicaltrials.gov/ct2/show/NCT05897619

**International Registered Report Identifier (IRRID):**

PRR1-10.2196/59461

## Introduction

Maternal mental health plays a pivotal role in influencing the health and well-being of both mothers and their children. In the United States, there is a growing maternal health crisis, in which mental health conditions rank among the top causes of preventable pregnancy-related deaths for mothers [1,2]. During pregnancy and the postpartum period, perinatal mood and anxiety disorders (PMADs) significantly influence a mother’s ability to function, care for, and bond with their newborn, which can have an impact on the child’s subsequent socioemotional developmental trajectory [3-5].

Though PMADs remain prevalent, with global perinatal depression rates at 26.3%, there is a serious lack of effective, accessible, and affordable mental health care for individuals in the perinatal period [6]. This is particularly true in low- and middle-income communities and countries. In these areas, a general scarcity of maternal mental health services is further exacerbated by stigma, fewer financial resources, and logistical barriers that interfere with connecting to care [7]. As such, many struggling individuals suffer in silence, without appropriate recognition or intervention for their pressing maternal mental health needs [8].

Fortunately, the digital age offers a beacon of hope in bridging the divide between growing community needs and barriers to care, and digital health interventions have the potential to improve maternal mental health outcomes [9]. Specifically, smartphone apps may be able to offer accessible and user-friendly platforms that teach useful coping strategies, which may be especially beneficial for mothers who have difficulty accessing traditional mental health services [10]. However, the landscape of mobile health apps using evidence-based information or psychological interventions is relatively sparse, and efforts to tailor these tools to meet the unique needs of the perinatal population are in the early stages of the field [11,12]. There are numerous mobile health apps targeting this population, with a range of tools for prevention, screening, and treatment, and varying content that includes psychoeducation, symptom monitoring, cognitive behavioral therapy (CBT), mindfulness, and attention bias modification training. Thus far, evidence regarding the effectiveness of existing mobile health interventions is mixed. Recent meta-analyses and systematic reviews demonstrate that app-based interventions are effective for individuals with mild-to-moderate symptoms of perinatal depression and anxiety and may be effective at preventing and treating postpartum depression compared to control groups [12,13]. Few of the existing apps use both evidence-based strategies and provide psychoeducation, as well as target both pregnant and postpartum individuals and both depression and anxiety. More research is needed to aid in the development of technology that is feasible, acceptable, and effective for pregnant and postpartum individuals who need tools to support their mental health.

To help address the critical gap in care needs, this study will present a protocol to assess the feasibility and acceptability of the novel Perinatal Skills Program within Maya, a cognitive-behavioral skills app. Maya-Perinatal Skills Program (M-PSP) is a 12-session mobile app–based program teaching evidence-based strategies to manage mood and anxiety symptoms for perinatal individuals. Following a community-based participatory research model, the study team is seeking feedback from users throughout the early stages of program development. User input will help inform future iterations of the app-based program in hopes of creating a tool that can effectively meet the needs of this population. This protocol represents the first step in the development of a tool designed to increase access to evidence-based skills for individuals with PMADs.

## Methods

### Aims

The primary aim of this study is to assess the feasibility and acceptability of M-PSP. Specifically, the team will evaluate the mobile app–based program in terms of user satisfaction, perceived use of the program, and engagement with the program. A secondary aim of the study is to look for any associations between program completion and engagement with mental health symptoms.

### Design

This study is a mixed methods open pilot trial assessing the feasibility, usability, and acceptability of M-PSP in a perinatal population. There are no comparison arms in the trial. Outcomes will include a combination of quantitative and qualitative data gathered via self-report questionnaires, interviews, and data passively collected through the app. This methodology will allow for the collection of symptom data, participant perceptions and feedback on their experience using the app, and information on app use and engagement. Results will provide information on the potential use of this program in helping pregnant and postpartum individuals manage mood and anxiety symptoms, as well as future directions for program development.

### Setting

The study is being conducted through a large academic medical center in New York City. Participants will be recruited through various departments within the medical center, including a psychiatry specialty center, primary care and pediatric settings, and obstetrics and gynecology clinics. These sites range in providing services through private pay, private insurance, or public insurance.

### Participants

The study is currently open to recruitment, with the aim to recruit 50 pregnant or postpartum individuals across all sites. In prior studies, feasibility studies often include a sample size of approximately 50 participants, which is deemed appropriate to ensure robust findings and comprehensive insights into app usability and effectiveness, balancing the need for inclusivity and accurate representation while accounting for potential participant attrition in digital mediums [14,15]. The inclusion criteria for study participation include (1) aged 18 years or older; (2) pregnant or up to 12 months post partum; (3) scoring 8-16 on the Edinburgh Postnatal Depression Scale (EPDS) [16] or 5-14 on the Generalized Anxiety Disorder-7 Scale (GAD-7) [17]; (4) able to read and speak English; (5) have access to a smartphone or mobile device capable of receiving SMS pushes and supporting Qualtrics Surveys and App (Weill Cornell Medicine); and (6) available to speak by phone or secure videoconference. The exclusion criteria include (2) safety concerns at the time of enrollment; (2) current substance use disorder; (3) current symptoms of psychosis or mania; and (4) history of bipolar or psychotic disorder.

M-PSP is designed to address mild-to-moderate anxiety and depression symptoms and may not be appropriate for more severely impaired individuals. In order to secure feedback from participants matching the app’s target population, the inclusion criteria include those scoring in the mild-to-moderate range on anxiety and depression ratings. Participants with safety concerns at the time of intake, including recent suicidal ideation or self-harm, are excluded as they are at higher risk for heightened symptoms and risk in the perinatal period. Eligibility is not impacted by receipt of clinical care outside of the study, and participants are not excluded if they are receiving concurrent treatment for depression or anxiety.

Study participants may be disenrolled from the study if they are nonresponsive to 16 outreach attempts across 4 consecutive weeks or if their pregnancy ends.

### Outcome Measures

#### Primary Outcome Measures

##### Feedback Interviews

Feedback interviews, led by staff via phone or video calls, include open-ended questions aimed to elicit qualitative feedback on program content, structure, and functionality, as well as user engagement and satisfaction. Questions such as “What ways could the app have been better tailored to help expectant or postpartum individuals?” will be asked to have a better gauge of acceptability, while questions like “What suggestions or comments do you have about the total number of sessions?” help address the feasibility of an intervention during and after pregnancy.

##### In-App Surveys

Weekly in-app feedback surveys are self-report measures that include Likert-scale questions regarding participant experience with specific session-by-session app-based content and structure (eg, “How helpful was Cognitive Practice [TINTED or Thinking Like a Lawyer]?”; “How likely are you to review any part of Session 3 again [eg, video, exercises, or quiz]?”). In particular, questions such as “How helpful was the content for you as an individual who is pregnant or postpartum?” and “How easy was it to fit using the app into your day-to-day life?” inform us of the acceptability and feasibility of the app, respectively, throughout the study.

##### User Version of the Mobile Application Rating Scale

Whereas the in-app surveys elicit session-by-session feedback, the user version of the Mobile Application Rating Scale (uMARS) elicits more global feedback on the overall app experience. The uMARS [18] is a validated 27-item self-report questionnaire evaluating engagement, functionality, aesthetics, information, subjective quality, and perceived impact of the app-based program. In particular, engagement, aesthetics, and information subscores will be used to measure acceptability and the functionality subscale will reflect the app’s usability.

Mean scores are derived for each of these subsections, which can then be averaged to determine an overall App Quality Score. Responses are rated between 1 and 5, with 5 indicating higher levels of satisfaction. Stoyanov et al [18] found that uMARS exhibited satisfactory internal consistency (α=.90).

##### Passive Data Collection

M-PSP passively collects data, encompassing information captured without active user input, such as when sessions were completed, frequency of program usage, and program completion. These data points will serve as measures of program feasibility and user engagement.

### Secondary Outcome Measures

#### Intake Survey

Participants will complete an intake questionnaire comprising questions related to demographics, health, mental health, and pregnancy history.

#### Edinburgh Postnatal Depression Scale

The EPDS [16,19] is a 10-item self-report measure of mood symptoms in perinatal individuals. Responses are coded on a scale of 0=lowest severity to 3=highest severity, with 30 being the highest score possible. Cutoffs are as follows: scores 7 and below indicate depression is unlikely, scores 8-13 indicate mild depression, 14-18 indicate moderate depression, and 19 and higher indicate severe depression [20]. Cox et al [21] found that the EPDS is a valid screening scale for depression and has satisfactory sensitivity (79%) and specificity (85%).

#### The Edinburgh Anxiety Subscale

The Edinburgh Anxiety Subscale [22] uses items 3, 4, and 5 from the EPDS to assess levels of anxiety among perinatal individuals. A score of 5 and above indicates the presence of anxiety [23].

#### Pregnancy Symptom Tracker

The daily Pregnancy Symptom Tracker (PST) is an ecological momentary assessment tool that aims to merge and consolidate gold-standard symptom measures into a format that is friendly and efficient for users, while also tracking information related to sleep, exercise, and daily behaviors. Developed by a group of psychiatrists and reproductive psychiatrists, the measure takes 30-60 seconds to complete. Users use a sliding scale to indicate the degree of each symptom or behavior present within the last 24 hours.

#### GAD-7

The GAD-7 [17] is a 7-item self-report measure assessing symptoms of anxiety. Responders indicate the frequency of symptoms on a scale of 0=not at all to 3=nearly every day, with total scores running from 0 to 21. A score of 0-4 indicates minimal anxiety, 5-9 indicates mild anxiety, 10-14 indicates moderate anxiety, and 15-21 indicates severe anxiety. Spitzer et al [17] found that the GAD-7 exhibited reliable sensitivity (89%) and specificity (82%), validating its effectiveness as a screening tool for generalized anxiety disorder.

#### Patient Health Questionnaire-9

The Patient Health Questionnaire-9 (PHQ-9) [24] is a validated 9-item self-report measure assessing symptoms of depression. Responders indicate the frequency of symptoms on a scale of 0=not at all to 3=nearly every day. Scores can range from 0 to 27, with an additional qualitative question regarding the level of difficulty caused by symptoms ranging from no difficulty at all to extreme difficulty. Scoring interpretation is as follows: 1-4 (minimal depression), 5-9 (mild depression), 10-14 (moderate depression), 15-19 (moderately severe depression), and 20 and greater (severe depression). Kroenke et al [24] found that a PHQ-9 score ≥10 exhibited a satisfactory sensitivity of 88% and a specificity of 88% for major depressive disorder.

#### Perinatal-Post Traumatic Stress Disorder Questionnaire-II

The Perinatal-Post Traumatic Stress Disorder Questionnaire-II (PPQ-II) [25] is a validated 14-item self-report measure assessing symptoms of trauma among postpartum individuals. Frequency and duration of symptoms are rated on a 5-point Likert scale from 0=not at all to 4=often for more than a month, with total scores ranging from 0 to 56. Scores of 19 above indicate a clinical range of symptoms that would benefit from additional support. Komurcu Akik and Durak Batigun [25] found that the PPQ-II demonstrates validity and reliability as a scale for assessing the perinatal post-traumatic stress symptoms experienced by mothers.

### The M-PSP Intervention

The Perinatal Skills Program was developed within Maya*,* a CBT skills app that is customizable and allows for the creation of individualized programs for multiple populations. M-PSP was adapted from one such program, Maya-Cognitive Behavioral Skills Program (M-CBT), which was a 12-session program that provided gold-standard, evidence-based strategies to help young adult users learn tools to manage anxiety and low mood. Preliminary data from a feasibility and acceptability trial of M-CBT suggest that the app is feasible and acceptable, and results showed a substantial decrease in both depression and anxiety from pre- to post-intervention [26]. In response to rising maternal mental health needs, an expert team of clinical psychologists specializing in CBT and reproductive psychiatrists with expertise in perinatal mental health adapted M-CBT into M-PSP. The team also consulted with physicians within pediatric primary care and obstetrics departments to better understand the needs of expectant and new parents. Changes to the original program included tailoring language and examples to address concerns that are common during and after pregnancy, as well as modifying skills as appropriate for the perinatal population. Several new skills were added, such as tools for managing communication and relationships as an expectant or new parent (Multimedia Appendix 1).

Users can work through each M-PSP session on their own schedule and are able to unlock up to 2 sessions per week. This pacing mirrors typical CBT session structure, allowing time for homework and practice between sessions, which is crucial for skill development and mastery [27,28], and acknowledges research showing that most people engage with apps for a limited duration, necessitating a structured yet flexible format to maximize engagement and effectiveness [29,30]. Each session in the program includes 3 main sections: learn (where new information and skills are shared), practice (where users can try out the skills they learned), and review (wrap-up of key lessons and takeaways). Users are introduced to a range of evidence-based tools throughout the 12 sessions (Table 1). There are also homework assignments for each session allowing users to practice what they have learned throughout the week. Users can individualize their experience by entering and tracking mood and anxiety ratings, planning for real-world skills use, personalizing examples, choosing preferred exercises to try, and reflecting on skill practice. The app has been programmed to help users see their progress through the app and track change toward self-identified goals. Users are also able to make use of tabs within the app for quick access to specific information, such as videos, skill practice, and favorite tools. Study staff have weekly meetings to ensure protocol is adhered to.

**Table 1:** M-PSP^a^ session content.

Session	Session objectives and content
Session 1: Understanding symptoms and treatment	Psychoeducation on perinatal anxiety and mood symptoms Sleep tips for pregnancy and post partum. Identifying common perinatal patterns of thoughts, feelings, and behaviors
Session 2: Facing challenges	Identifying how emotions impact behavior during the perinatal period Learning to gradually approach and overcome challenging tasks “Face It!” exercise to craft an individualized goal ladder for daily practice
Session 3: Shifting thoughts	Cognitive restructuring techniques to reframe unhelpful thinking Guidance on effective communication and managing evolving relationships Introduction of “TINTED Thoughts” exercises to recognize and correct cognitive distortions
Session 4: Relaxing your body and mind	Introduction to relaxation exercises and audio-guided muscle relaxation meditation session. Distress tolerance tips to manage stressful moments “Face It!” exercise to craft an individualized goal ladder for daily practice
Sessions 5-10: Practice and use skills	Revisiting the list of challenging situations and tracking progress Continued practice of helpful thoughts and “Face It!” exercises Introduction to problem-solving skills and additional distress tolerance exercises
Sessions 11-12: Maintenance and relapse prevention	Guided selection of an exercise for ongoing skill practice Skill review and reflection exercise Guidance on next steps to support sustained mental health

^a^M-PSP: Maya-Perinatal Skills Program.

### Procedure

As of October 2024, the study is open to recruitment. Recruitment strategies involve advertisements through listservs, clinician information sessions, and flyers within inpatient and outpatient departments. Interested individuals can complete a digital contact information form via a Health Insurance Portability and Accountability Act–compliant survey platform or have their providers complete the contact forms on their behalf.

Study staff reach out to individuals who have completed the form to screen for eligibility. Eligible participants are given further details about study involvement and complete informed consent. Study staff help participants download the app and provide an orientation, with recommendations to use the program for 10 minutes a day, 4 days a week. While it is recommended to complete the program within 6 weeks, sessions can be completed at the participant’s convenience. All participants will be allowed up to 18 weeks for completion in order to account for delays or interruptions that may have occurred during pregnancy or after labor and delivery. Within 1 week of the study orientation, the research assistant will conduct a brief 5-minute phone check-in using a scripted protocol to ensure participants have been able to access and start the app, check on potential technical issues, and answer any questions related to app navigation.

Throughout participation, participants will be able to complete up to 2 sessions per week and will complete the in-app feedback surveys after each session. They will also be sent a text to their phone with a link to complete the daily PST. Additional assessments occur at midpoint (following completion of session 6), post (completion of session 12), and follow-up (6 weeks after post). These assessments include the staff-led feedback interviews via phone or videoconference, as well as the uMARS and all symptom measures. When risk items are endorsed on the daily PST, symptom measures, or during the assessments, study staff are notified and the participant is contacted by a licensed provider to conduct a formal risk assessment and safety plan as necessary.

To aid in retention and study completion, study staff monitor app completion progress and maintain contact with participants to schedule feedback interviews (Table 2). Through these methods, staff are able to problem-solve technical difficulties and other barriers to session completion as needed. Participants are encouraged to reach out via email or phone with any questions or concerns. Study staff follow institutional review board (IRB) guidelines to report any adverse events within the required timeline.

**Table 2:** SPIRIT^a^ checklist.

	Prestudy	Intake	Weeks 1-6	Midpoint	Weeks 7-12	Post	6-week follow-up
Contact form	✓						
Eligibility screen		✓					
Informed consent		✓					
Download of Maya		✓					
Background questionnaire		✓					
App sessions			✓		✓		
In-app feedback			✓		✓		
Symptom measures		✓		✓		✓	✓
Daily symptom tracker			✓	✓	✓	✓	✓
Feedback interview and uMARS^b^				✓		✓	✓
Adverse event monitoring	✓	✓	✓	✓	✓	✓	✓

^a^SPIRIT: Standard Protocol Items: Recommendations for Interventional Trials.

^b^uMARS: user version of the Mobile Application Rating Scale.

### Data Analysis Plan

To analyze qualitative data from the feedback interviews, the grounded theory approach [31] will be used to review all responses and develop a consensus on existing themes. The data will then be systematically coded using these themes in order to identify core concepts and response patterns to derive meaningful interpretations of the feedback.

Data passively collected from the app will be transformed into practical means and ranges that will be used to understand user engagement and program feasibility. Means and SDs will be computed for demographic information and relevant symptom and acceptability questionnaires. Continuous scores from symptom scales and acceptability questionnaires will be analyzed in relation to app use and engagement. Correlations between these variables with be assessed using either Pearson or Spearman correlation depending on data distribution. For example, we will examine the correlation between EPDS symptom scores and app completion rates to explore whether postpartum depression symptoms are related to program feasibility. Hypothesis testing will be conducted with a *P* value=.05 as the threshold for statistical significance, and clinical significance will be considered for correlations that meet a meaningful cutoff (eg, 0.6 or 0.8). This analysis will help identify any demographic characteristics or symptoms that may require greater attention in future iterations of the program to ensure its effectiveness for a broad range of perinatal individuals. For participants who dropped out before study completion, all available data will be included, and any missing data will be handled using simple imputation. A correlation matrix will be generated to explore the relationships between symptom scales (eg, EPDS and GAD-7) and app engagement variables (eg, frequency of use, duration of use), allowing us to investigate possible associations without the need to correct for multiple testing in this exploratory pilot study.

### Ethical Considerations

The study was granted approval through the Biomedical Research Alliance of New York IRB (BRANY #23-02-134-380) and the internal IRB within the academic medical center at which the trial is being conducted. The study has been registered at ClinicalTrials.gov (protocol number NCT05897619). This protocol was written in accordance with the SPIRIT (Standard Protocol Items: Recommendations for Interventional Trials) guidelines (Multimedia Appendix 2) [32]. All participants provided informed consent and were made aware that they could withdraw from the study at any time without penalty. Participants are not compensated for their participation. All participants may continue to use the MAYA app following study completion if they so choose and will be given a list of resources, including referrals for clinical care.

While no individual data will be reported, the study team will report results via publication following trial completion. Health care providers within the Weill Cornell Medicine or NewYork-Presbyterian network may be given access to a summary of qualitative and quantitative outcomes. Every participant is given a unique study identifier. All data collected were confidential and safely stored per protocol approved by the IRB. As a small, unblinded study that does not involve invasive procedures or greater than minimal risk, there is no data monitoring committee. The study is internally sponsored, with no external funding organization involved in study design, management, monitoring, or analysis. The study is fully monitored by internal co-investigators, with IRB oversight through BRANY**.**

## Results

The pilot trial received internal funding in August 2023 and data collection started in September 2023. As of October 2024, 32 participants have been enrolled in the study. It is anticipated that enrollment will continue throughout 2024, with projected recruitment completion in December 2024. Data analysis will be completed upon study completion of the last participant.

## Discussion

This study aims to assess the feasibility, usability, and acceptability of the M-PSP, a 12-session program teaching skills derived from CBT and adapted to suit the unique needs of a perinatal population. Regarding feasibility, it is anticipated that outcomes will indicate the program is able to be worked through by users, but that pacing and session length may need more flexibility in future versions of the app to account for the particular demands of this population. It is hypothesized that users will find the skills to be acceptable, but that some of the sample videos need revision to more directly reflect mood and anxiety concerns during and after pregnancy. Further, the study team anticipates that users will have input on specific content that could be added to improve their satisfaction with the app. Regarding usability, it is anticipated that the app will be user-friendly and easy to work through. As the secondary aims are exploratory, there are no specific hypotheses related to symptom measures as they are related to engagement. These outcomes will inform future iterations of the program in hopes of developing a widely accessible and useful tool for pregnant and postpartum individuals.

While the findings from this pilot study will provide valuable insights, several limitations must be acknowledged. This study relies on self-report data, which may introduce biases such as social desirability or recall errors. In future studies, clinician assessment of symptoms could help to mitigate these concerns. In this and future studies, the use of passively collected app usage data should provide some objective metrics regarding engagement and feasibility. A further limitation is the small sample size. As a pilot trial, this study is not powered to detect statistical significance, as the primary aim is to assess feasibility and acceptability rather than efficacy. Therefore, these findings should be interpreted as preliminary, and further research with larger samples will be necessary to assess the program’s effectiveness in improving mental health outcomes.

Despite these limitations, this study is an important step in helping to fill the gap between an increasing maternal mental health crisis and barriers to appropriate support. By using a community-based-participatory research model, the development team has been able to consider input from researchers and clinicians across different specialties and will be able to incorporate feedback from end users through each stage of program development. Once feedback has been considered and incorporated into the program, the team will look to conduct future studies to assess the effectiveness of the tool in helping perinatal individuals manage anxiety and mood symptoms. Per guidelines on progressing from a pilot trial to a fully powered randomized controlled trial (RCT) [33], the research team has outlined a number of criteria that will influence the next steps. To ensure adequate participant numbers for an RCT, the pilot trial will need to demonstrate a 50% retention rate between screening and enrollment. The team will also move forward if 75% of participants complete primary outcome measures, which consist of feasibility, acceptability, and usability feedback interviews and surveys. While the study team will track adherence to the intervention (ie, completing M-PSP sessions), this will not be used as a criterion for progressing to a full RCT as nonadherence may be attributable to feasibility or acceptability concerns that the pilot study intends to illuminate so that future versions of the app can be modified prior to an RCT.

## Appendix

Multimedia Appendix 1 Sample screenshots of the MAYA Perinatal Skills Program.

Multimedia Appendix 2 SPIRIT (Standard Protocol Items: Recommendations for Interventional Trials) checklist.

## Abbreviations

CBT: cognitive behavioral therapy
EPDS: Edinburgh Postnatal Depression Scale
GAD-7: Generalized Anxiety Disorder-7 Scale
IRB: institutional review board
M-CBT: Maya-Cognitive Behavioral Skills Program
M-PSP: Maya-Perinatal Skills Program
PHQ-9: Patient Health Questionnaire-9
PMAD: perinatal mood and anxiety disorder
PPQ-II: Perinatal-Post Traumatic Stress Disorder Questionnaire-II
PST: pregnancy symptom tracker
RCT: randomized controlled trial
SPIRIT: Standard Protocol Items: Recommendations for Interventional Trials
uMARS: user version of the Mobile Application Rating Scale

## Data Availability

The datasets generated during this study will be available from the corresponding author on reasonable request.
