# Supplementary figures and images for: Feasibility of a Mobile App–Based Cognitive-Behavioral Perinatal Skills Program: Protocol for Nonrandomized Pilot Trial
Source: JMIR Res Protoc. 2025 Jan 28;14:e59461. doi: 10.2196/59461 (PMC11815301; doi:10.2196/59461)

**Multimedia Appendix 1**

Sample screenshots of the *MAYA* Perinatal Skills Program

**
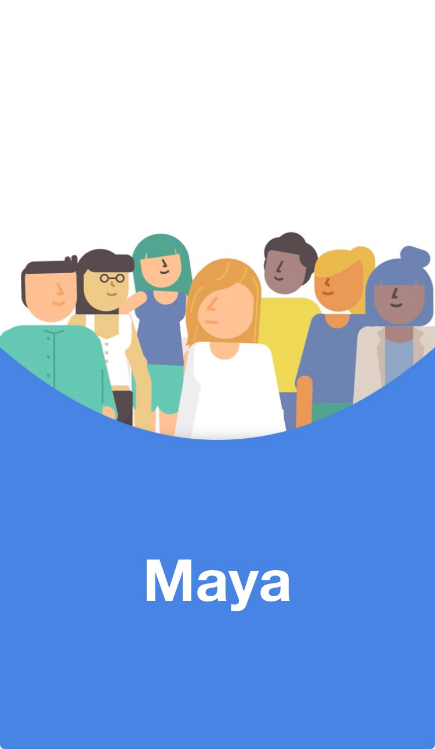
**
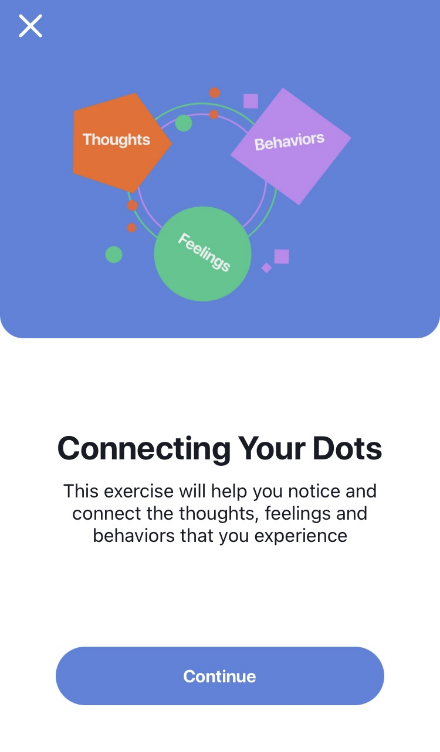

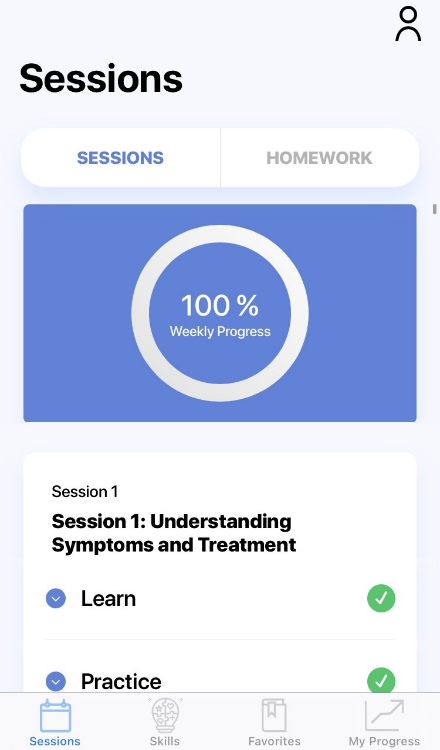


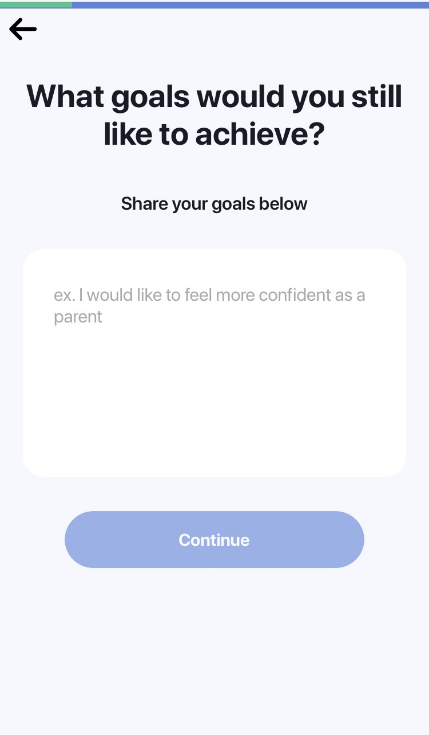

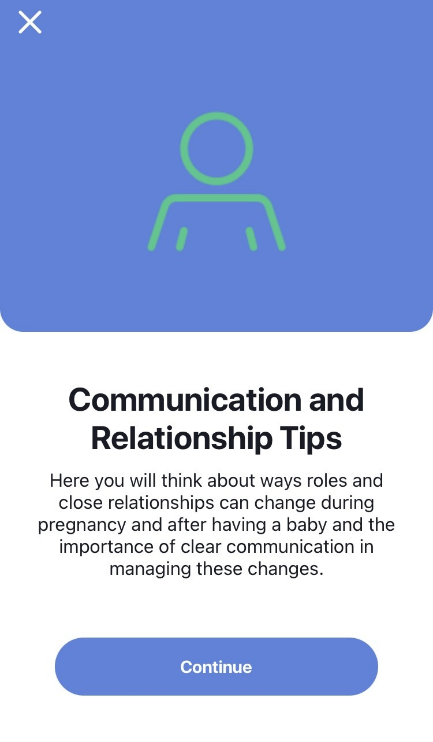

Supplement: Multimedia Appendix 1 [file resprot_v14i1e59461_app1.docx]
